# All-trans retinoic acid and fibroblast growth factor-2 enhance the fertility rate and embryo development in polycystic ovary syndrome mouse model

**DOI:** 10.22038/IJBMS.2024.70509.15328

**Published:** 2024

**Authors:** Auob Rustamzadeh, Maryam Anjomshoa, Narges Bahreini, Shahram Darabi, Mohammad Jafar Rezaie, Shohreh Rezaei, Mohammad Rahimi-madiseh, Fatemeh Deris, Saeed Zamani

**Affiliations:** 1 Department of Anatomical Sciences, School of Medicine, Iran University of Medical Sciences, Tehran, Iran; 2 Cellular and Molecular Research Center, Research Institute for Non-communicable Diseases, Qazvin University of Medical Sciences, Qazvin, Iran; 3 Department of Anatomical Sciences, Faculty of Medicine, Shahrekord University of Medical Sciences, Shahrekord, Iran; 4 Faculty of Medicine, Shahrekord University of Medical Sciences, Shahrekord, Iran; 5 Cellular and Molecular Research Center, Research Institute for Health Development, Department of Anatomical Sciences, Kurdistan University of Medical Sciences, Sanandaj, Iran; 6 Medical Plants Research Center, Basic Health Sciences Institute, Faculty of Medicine, Shahrekord University of Medical Sciences, Shahrekord, Iran; 7 Department of Epidemiology and Biostatistics, Shahrekord University of Medical Sciences, Shahrekord, Iran

**Keywords:** Embryo development, Fertility agents, Growth factor, In vitro oocyte maturation – techniques, Reproduction

## Abstract

**Objective(s):**

Polycystic ovary syndrome (PCOS) causes a developmental arrest of antral follicles and disrupts oocyte maturation. Retinoic acid (RA) and Fibroblast Growth Factor-2 (FGF2) are effective in follicle growth, thus their effects on histopathology and in vitro fertility of oocytes were investigated in PCOS-induced mice.

**Materials and Methods:**

Eighty female NMRI mice were randomly divided into 8 groups including 1-Normal mice, 2-PCOS mice without any treatment, 3-Normal mice treated with RA, 4-Normal mice treated with FGF2, 5-PCOS mice treated with RA, 6- PCOS mice treated with FGF2, 7- PCOS mice treated with RA and FGF2, and 8- Normal mice treated with RA and FGF2. Following PCOS induction, the mice were treated with intraperitoneal RA and FGF2 as a treatment. Then ovarian stimulation, for preparing the oocyte and embryo microscopic examinations was performed. After oocyte morphometry, through *in vitro* fertilization, the embryo formation was assessed. Data was analyzed by one-way ANOVA and Tukey tests.

**Results:**

The results showed simultaneous injection of RA and FGF2 into PCOS-induced mice increases antral follicles and corpus luteum, but decreases cystic follicles. Simultaneous injection of these two substances into healthy mice increases the pre-antral follicles and corpus luteum. Simultaneous injection of RA and FGF2 increases the number of embryos in both control and intervention groups.

**Conclusion:**

It can be concluded that RA and FGF2 increase the maturity of ovarian follicles, the number of two-celled embryos, and the number of grade-A embryos in mice with PCOS, which is more effective when these two substances are injected simultaneously.

## Introduction

Polycystic ovary syndrome (PCOS) is a pathological state that impacts females during their reproductive years. This endocrinological abnormality serves as the prevailing cause of infertility in the female population. The reported prevalence of this condition has been found to vary. According to the diagnostic criteria outlined by the National Institutes of Health (NIH), the prevalence ranges from 6% to 10%. However, when the Rotterdam criteria are employed, the estimated prevalence increases to 15%. In order to diagnose PCOS, it is necessary to meet two out of three specific criteria. These criteria include anovulation, hyperandrogenism, and the presence of polycystic ovaries as detected by ultrasound (1-3). It is a complex endocrinopathy, and its presentation has great diversity (4). Despite the severity of PCOS on women’s health, its pathogenesis and etiology are poorly known (3). Same as the etiology of irregular ovulation or anovulation, it remains challenging and debatable (5). There may exist multiple pathways that contribute to the occurrence of the anovulation phenomenon. Anovulation can be induced by either hypothalamic/pituitary conditions, primary ovarian disorders, or a combination of both. The characteristic feature of anovulation is the developmental arrest of many antral follicles. Consequently, these follicles are unable to progress to the preovulatory stage (5). 

Many different factors are involved in the follicular maturation process. Among these factors, fibroblast growth factors (FGFs) stand as one significant contributor. The production of FGFs is attributed to the granulosa and theca cells. FGFs are classified into four primary classes (FGF1-4), each of which plays a distinct role in various stages of folliculogenesis, such as the initiation of the primordial follicle, division of cumulus and granulosa cells, programmed cell death, and glycolysis. FGF2, also known as basic FGF, plays a crucial role in the process of oocyte maturation. Researchers declared that FGF2 stimulates follicles in the early stage of development (primordial follicles). However, the role of FGFs in the last period of oocyte maturation remains unclear (6, 7). Additionally, other investigations conducted on diverse species have suggested that retinoids exhibit efficacy during the initial phase of reproductive processes; this can include follicular development or oocyte growth. The healthy follicles have shown the highest concentration of retinol in bovine. Retinol injection in sheep has improved *in vitro* development of morula to the blastocyst stage. Although the mechanism of reproductive improvement through retinol administration is not completely known, affecting LH and FSH receptors, increasing the mRNA quality, and protection against oxidative stress have been proposed. Moreover, retinol exerts an impact on the expression of various growth factors, such as Midkine, which plays a role in stimulating the development of follicles, the maturation of cytoplasm, and the proliferation of oocytes (8).

The utilization of various animal models will undeniably expedite the comprehension of the pathogenesis of PCOS, and possibly lead to the development of new therapeutic protocols for treating and preventing PCOS in women (3). It is proposed that the treatment of PCOS with both FGF2 and retinol will be more effective than a single therapy in PCOS-induced animals. In this study, mice were used to induce PCOS, hence they are affordable and facile to handle, their reproductive cycles are shorter, and their genetic backgrounds are stable. Retinoic acid (RA) and FGF2 were administered for assessment of the folliculogenesis and embryo development of mice with induced PCOS.

## Materials and Methods

PCOS-like phenotype was first induced in the female mice and then their treatment with RA and FGF2 was started. After ovarian stimulation, the oocytes were collected and assessed in two steps: first microscopic examination was done. Second, they were fertilized with sperm to evaluate their quality after treatment. More details are as follows. 

***Animals***

In this experiment, the ARRIVE guidelines are followed. The animals in this study were 80 female NMRI mice aged about 4 weeks, weighing 30 g, and were purchased from Tehran Pasteur Institute, Iran. All procedures were carried out in accordance with the regulations of the University and the Guide for the Care and Use of Laboratory Animals of the National Institutes of Health (Ethics code: IR.SKUMS.REC.1396.253) and Guide for the Care and Use of Laboratory Animals (8th edition, National Academies Press). Full efforts were made to reduce the use of animals and to advance their welfare. The mice were divided into 8 groups (n=10) by simple randomization. The animals were placed in appropriate laboratory conditions (21±2 °C, 12 hr/12 hr light/dark, and good ventilation). The mice were monitored for distress, transient mild pain, or unexpected events through regular procedures. The study was blind, so, the investigator who reported the results was not aware of the mice`s grouping. Animal groups included:

1- Normal control group: did not receive any treatment.

2- Positive control group: received 40 mg/kg, intraperitoneal estradiol valerate injection.

3- RA control group: received 0.5 µg/µl, intraperitoneal RA injection.

4- FGF2 control group: received 10 µg/kg intraperitoneal FGF2 injection.

5- RA interventional group: mice with induced PCOS received 0.5 µg/µl, intraperitoneal RA injection.

6-FGF2 interventional group: mice with induced PCOS received 10 µg/kg, intraperitoneal FGF2 injection.

7. RA+ FGF2 intervention group: mice with induced PCOS received intraperitoneal injection of RA at a dose of 0.5 µg/µl, and FGF2 at a dose of 10 µg/kg.

8- RA + FGF2 control group: normal mice received intraperitoneal injection of RA at a dose of 0.5 µg/µl, and FGF2 at a dose of 10 µg/kg.

***Induction of polycystic ovary syndrome***

In order to induce PCOS, the mice in interventional groups received 40 mg/kg intraperitoneal estradiol valerate as a single dose injection (9). Followed by confirmation of PCOS induction, ovarian histology, and vaginal smear examination. Three mice in each group (24 mice) were anesthetized with ketamine hydrochloride (50 mg/kg) and xylazine (10 mg/kg), then executed by cervical dislocation, in accordance with ethical rules (according to the regulations of the University and the Guide for the Care and Use of Laboratory Animals of the National Institutes of Health (Ethics code: IR.SKUMS.REC.1396.253). Next, the ovaries were separated from the twisted oviduct tubes and placed in a PBS solution. The excess fat was carefully removed under a microscope, and the ovaries were placed in paraformaldehyde for histopathological procedures (10). The estrous cycle of the live mice in each group was evaluated by taking their vaginal smear, every day at the same time (7:00 am) for 14 days. Acyclicity or a low number of estrous cycles during 14 days and a low number of corpora lutea in ovarian histological samples were considered PCOS-like phenotypes. 

***RA and FGF2 treatment***

In interventional groups, after confirming the PCOS induction, treatment was started. A dose of 0.5 µg/µl body weight RA was injected intraperitoneally three times a week for 4 weeks (11). Intraperitoneal FGF2 at 10 µg/kg dose, three times a week for 4 weeks was injected (12).

***Ovarian stimulation***

After the last dose of FGF2, ovarian stimulation was triggered with 10 IU of pregnant mare serum gonadotropin (PMSG) which is injected intraperitoneally to stimulate ovulation. After 48 hr, 10 units of hCG were injected intraperitoneally. 

***Oocyte and sperm preparation***

Fifteen hr after hCG injection, the mice were deeply anesthetized with ketamine hydrochloride (50 mg/kg) and xylazine (10 mg/kg). Afterward, they were executed by cervical dislocation, to remove their fallopian tubes and transfer those into mineral oil. The fallopian tubes were immobilized using fine forceps. By making a delicate incision in the ampulla area, the oocyte is ejected. Next, the oocytes and cumulus cells around them were transferred into HTF (human tubal fluid) medium and kept in a CO_2_ incubator. 5 male mice were used to prepare sperm. After ethical execution, 5 μl of sperm was removed by sampler, as it was 1 × 10^6^ sperm per milliliter. The sperm sample was stored in a 5% CO_2_ incubator for 5 hr. After preparation for the oocyte, two more steps were followed: 1. an immediate microscopic examination was done, 2. After 6 hr, when the oocytes reached the metaphase II stage, the IVF technique was performed.

***Microscopic examination***

For microscopic examination, oocytes were placed in hyaluronidase solution for one minute to separate the granulosa cells from the surrounding area. To observe the quality, number, and maturation, they were inspected by a stereo microscope (13).

***Oocyte morphometry***

After taking images with a reverse microscope, they were analyzed by Image J software. The oocytes were evaluated for cytoplasmic diameter, zona pellucida (ZP) thickness, and perivitelline space diameter. The longest oocyte diameter was considered for cytoplasm diameter morphometry and thickness of ZP. For estimating the ZP thickness, the thickness of both sides was added together and their sum was considered as the actual thickness. Furthermore, the diameter of the space around the yolk was estimated by subtracting the cytoplasm diameter and ZP thickness from the total diameter.

***IVF technique***

First, metaphase II oocytes (the presence of the second polar body indicates metaphase II oocytes) were placed on a plate containing culture medium plus liquid paraffin oil and kept in an incubator at 37 °C. After one hour, the sperm was inoculated. A sperm suspension with a concentration of 50,000/ml was prepared and added to the metaphase II oocytes in the KSOM medium. The oocyte plate was placed in an incubator. After 5 hr, the fertilization process was examined under a microscope (14).

***IVF process evaluation***

The IVF process was evaluated by observing the morphology of two-celled embryos by a stereomicroscope. The embryo quality was scored on four scale systems (Bolton) as follows (15).

Embryos have uniform and round blastomeres without any fragmentation, smooth cytoplasm, and clear yellow ZP.

Blastomeres have almost unequal size, about 10% fragmentation, and some granules in the cytoplasm. 

Blastomeres have completely unequal size, more than 50% fragmentation, and large granules and vacuoles in the cytoplasm. 

Blastomeres have a completely unequal size, almost complete fragmentation (more than 80%), and the cytoplasm is occupied with large and numerous vacuoles. 

Overall, blastomeres in grade A have the least damage, while in grade D, the most morphological changes and the worst damage have occurred.

***Statistics analysis***

SPSS (version 19) was used to conduct one-way ANOVA and Tukey tests for inter-group comparison. *P*<0.05 was defined as statistical significance. 

## Results

***Oocyte morphometry***

**Figure 1:**
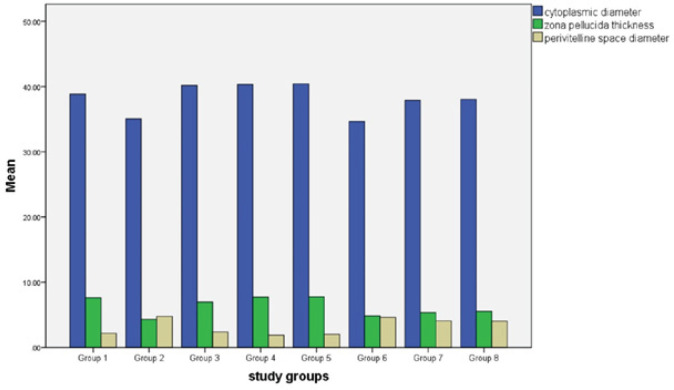
Effects of RA, FGF2, and both on cytoplasmic diameter, zona pellucida thickness, and perivitelline space diameter in study groups. The significant change in these parameters was observed in group 2 compared to group 1. When comparing group 7 to group 2, there was a significant increase in cytoplasmic diameter; in group 8, this increase was even greater. ZP thickness was significantly higher in groups 7 and 8 than in group 2, and perivitelline space diameter was significantly lower in groups 7 and 8 than in group 2

Based on the results of the present study, cytoplasmic diameter, ZP thickness, and perivitelline space diameter had a significant decrease in group 2 compared to group 1 (*P*<0.0001). Cytoplasmic diameter in the seventh group was increased significantly, in comparison with group 2 (*P*=0.013), this increase was even more in group 8 (*P*=0.007). Also, oocyte cytoplasmic diameter in groups 7 and 8 was significantly increased compared to group 6 (*P*=0.003 and *P*=0.001). ZP thickness in groups 7 and 8 had a significant increase in comparison with group 2 (*P*=0.012 and *P*=0.001) and perivitelline space diameter in groups 7 and 8 had a significant decrease compared to group 2 (*P*=0.017 and *P*=0.009) (Figure 1). 

***Estrous cycle***

The estrous cycle was assessed during 2 consecutive weeks by taking a vaginal smear in the study groups. Then, the estrous cycle between the control group and the positive control group was compared. In both, the mice had regular 4 to 5-day estrous cycles, including Metestrus, Proestrus, Estrus, and Diestrus. Acyclicity or a low number of estrous cycles during 14 days and a low number of corpora lutea in ovarian histological samples were considered PCOS-like phenotypes. 

***Follicles at different stages of development***

**Figure 2:**
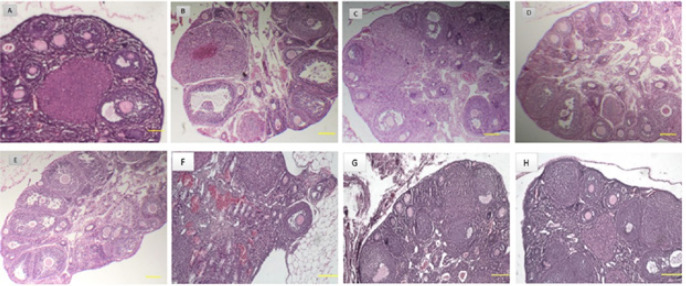
Morphological assessment of tissue sections prepared from ovaries in the studied groups focusing on follicular growth and stroma integrity

By examining the morphology of tissue sections prepared from ovaries in the normal control group (group 1) (Figure 2A), follicles in different stages including primordial follicles (PMF), primary follicles (PF), preantral follicles (PAF), antral follicles (AF), and a significant number of corpus luteum were observed. In the ovaries of other groups, a variable number of follicles at different stages of development as well as cystic follicles with a very thin layer of granulosa cells were observed (Table 1). Regarding PMF, no statistically significant difference was observed between the study groups (*P*>0.05). The results showed that in the positive control group (group 2) after a single dose injection of estradiol valerate, the number of PAF and CF (cystic follicle) increased compared to the normal control group (group 1) (*P*˂0.05), but the number of CL** (**corpus luteum) has decreased (*P*˂0.05) (Figure 2B). Also, after injection of 0.5 μg/μl body weight RA in healthy mice (group 3), PAF increased compared to the normal control group (group 1) (*P*˂0.05) (Figure 2C). Injection of 10 µg/kg FGF2 body weight in group 4, increased PAF and CL compared to the normal control group (group 1) (*P*˂0.05) (Figure 2D). In group 5 (PCOS-induced mice), injection of 0.5 μg/μl body weight RA caused a significant increase in PAF (*P*˂0.05, Figure 2E). While after FGF2 injection at 10 µg/kg body weight to PCOS-induced mice (group 6), the number of AF and CL increased (*P*˂0.05), but CFs decreased in comparison to group 2 (*P*˂0.05) (Figure 2F). The results showed that simultaneous injection of RA and FGF2 into PCOS-induced mice (group 7) increased AF and CL compared to group 2 and decreased CFs (*P*˂0.05) (Table 1 and Figure 2G). In group 8 (healthy mice), co-injection of RA and FGF2 increased the number of PAFs and CL compared to normal control group 1 (*P*˂0.05), which indicates the high effectiveness of simultaneous injection of RA and FGF2 into healthy mice in increasing ovarian follicle maturation (Figure 2H).

***Comparison of in vitro fertility results***

Statistical analysis showed that the number of two-celled embryos in the positive control group (group 2) was significantly different from the normal control group (group 1) (*P*˂0.05). This indicates the negative effects of estradiol valerate on embryo survival. The results also showed that the number of embryos was the highest in the RA+FGF2 control group (healthy mice) and the lowest in the PCOS-induced mice group that received RA (0.5 μg/μl body weight). Also, PCOS-induced mice that received RA (0.5 μg/μl body weight) and FGF2 (10µg/kg) (group 7) had an increment in the number of two cell embryos compared to the positive control group (group 2) (*P*˂0.05). There was no significant difference between the other groups (Table 2).

***Evaluation of the quality of two-celled embryos***

Qualitative studies showed a significant decrease in the number of A-grade embryos and a significant increase of C and D-grade embryos in the positive control group (group 2) compared to the normal control group (group 1) (*P*˂0.05, Table 3). The results showed that grade-A embryos in PCOS-induced mice treated with RA (0.5 μg/μl body weight) (group 5), FGF2 (10 µg/kg FGF2 body weight) (group 6), and RA (0.5 µg /μl body weight) plus FGF2 (10 µg/kg FGF2 body weight) (group 7) had a significant increase compared to group 2 (*P*˂0.05). Also, in group 6, a significant decrease in grade C and D embryos was observed compared to group 2 (*P*˂0.05). In addition, simultaneous injection of RA and FGF2 into PCOS-induced mice (group 7) reduced C and D-grade embryos in comparison to group 2 (*P*˂0.05). Finally, in group 8 (healthy mice received RA plus FGF2), a significant increase in grade-A embryos was seen compared to group 1 (*P*˂0.05).

(A) Tissue sections prepared from ovaries in the normal control group (group 1). (B) Effect of single-dose estradiol valerate injection in the positive control group (group 2). (C) Effect of 0.5 μg/μl body weight single dose retinoic acid injection in healthy mice (group 3). (D) Effect of 10 µg/µl body weight FGF2 injection on healthy mice (group 4). (E) Effect of 0.5 μg/μl body weight single dose retinoic acid injection to polycystic ovary mice (group 5). (F) Effect of 10 µg/µl body weight FGF2 injection to mice with polycystic ovaries (group 6). (G) Effect of simultaneous injection of RA and FGF2 in PCOS mice (group 7). (H) Effect of simultaneous injection of RA and FGF2 in healthy mice (group 8). In all images, the scale bar quals 100 µm and the magnification is equal to 200, except in group 1 where it is 400.

**Table 1:** When study groups’ follicles at various developmental stages were compared, group 4 had higher PAF and CL than group 1. Group 5 showed a noteworthy rise in PAF. In contrast, group 6 experienced an increase in AF and CL numbers

**Groups** **(n=7)**	**Follicle development stage**				
	**Primordial**	**Preantral**	**Antral**	**Cystic**	**Corpus luteum**
**1**	26.8±0.71	6.4±0.52	5.2±0.24	1.2±0.2	15.7±0.47
**2**	26.2±0.82	13.9±0.72^a^	6.4±0.45	10.4±0.52^a^	3.4±0.3^a^
**3**	26.6±0.33	10.1±0.48^b^	9.6±0.4	1.5±0.16	17.4±0.54
**4**	26.8±0.25	9.4±0.71^c^	5.9±0.34	1.2±0.24	17.9±0.64^c^
**5**	24.8±0.44	9.3±0.66^e^	5.9±0.48	9.2±0.38^e^	4.5±0.42
**6**	25.2±0.14	13.7±0.8	9.3±0.33	8±0.44^f^	6.3±0.33^f^
**7**	25.2±0.74	15.5±0.58^d^	10.3±0.55	5.7±0.42	19.5±0.3^d^
**8**	26±0.44	14.2±0.55	9.5±0.34^g^	0.8±0.24^g^	8.5±0.45^g^

a, b, c, and d show a significant difference with group 1 (*P*<0.05); e, f, and g also show a significant difference with group 2 (*P*<0.05). Data are shown as MEAN±SEM.

**Table 2:** Comparison of in vitro fertility results in study groups indicates the negative effects of estradiol valerate on embryo survival. The number of embryos was the highest in the group 8 and the lowest in the PCOS-induced mice group 5. Also, PCOS-induced mice in group 7 had an increment in the number of two-cell embryos compared to group 2

**Groups**	**Total MII oocytes insemination**	**20–26 hr after IVF**	**30 hr after I** **VF**
		**2-cell embryo**	**dead cell**
**1**	322	11.3±0.47	2.2±0.38
**2**	315	6.1±0.4^a^	9.9±0.37^a^
**3**	286	11.1±0.6	2.6±0.4
**4**	295	12±0.36	1.9±0.27
**5**	296	5.8±0.44	10.3±0.57
**6**	294	7.2±0.35	9.2±0.35
**7**	283	9±0.49^b^	7.1±0.5^b^
**8**	294	13.4±0.6	1.4±0.3

a indicates a significant difference with group 1 (*P*<0.05), and b indicates a significant difference with group 2 (*P*<0.05). Data are shown as MEAN±SEM.

**Table 3:** Qualitative assessment based on the Bolton scale reveals that A-grade embryos and a significant increase in C and D-grade embryos in group 2 compared to group 1. grade-A embryos in PCOS-induced mice group 5, group 6, and group 7 had a significant increase compared to group 2

** Grade**	**A**	**B**	**C**	**D**
**Group**				
**1**	71±1.52	16.66±0.8	10±1.52	9.6±0.88
**2**	39.33±0.66^*^	12±2.08	20±2.3^*^	33.6±1.2^*^
**3**	70±1.15	11±1.15^*^	6.66±1.76	7.66±0.33
**4**	77.33±2.84	10±0.57^*^	5.6±0.66	5.33±0.8
**5**	53±2.08^#^	11.6±0.88	13.3±2.3	20.6±2.18^#^
**6**	59.6±0.8^#^	10.33±0.6	10.3±0.88^#^	17.6±2.02^#^
**7**	65.33±1.3^#^	8.66±0.6	5.33±0.3^#^	15±2^#^
**8**	80±1.15^*^	7.33±0.6^*^	5.6±0.66	5±1.73

*Indicates a significant difference with group 1. # indicates a significant difference with group 2. Data are shown as MEAN±SEM. (Grade of Bolton scale: A. Embryos have uniform and round blastomeres without any fragmentation, smooth cytoplasm, and clear yellow Zona pellucida. B. Blastomeres have almost unequal size, about 10% fragmentation, and some granules in the cytoplasm. C. Blastomeres have completely unequal size, more than 50% fragmentation, and large granules and vacuoles in the cytoplasm. D. Blastomeres have completely unequal size, and almost complete fragmentation (more than 80%), and the cytoplasm is occupied with large and numerous vacuoles.

## Discussion

The particular characteristic of anovulation in PCOS is characterized by the existence of a multitude of follicles that cease to progress in the antral stage (with a diameter range of 2–8 mm) and fail to develop. Consequently, there is an absence of a dominant follicle. Some studies suggest that the failure of antral follicles may be attributed to the initial phases of folliculogenesis (2). Therefore, this study focused on the early stage of follicle growth. On the other hand, many studies have indicated the great effects of RA or FGF2 on oocyte development, especially during *in vitro* maturation (IVM). Considering the importance of the early stage of follicular growth as the root of its stop, we designed to observe the *in vivo* effects of FGF2, RA, dual RA, and FGF2 injections at a very early stage, in mice with induced PCOS. Besides, their effects on the future fertilization of mature oocytes into two-cell stage embryos were studied.

In this study, after injection of RA and FGF2, the oocyte cytoplasmic diameter increased in PCOS-induced mice and it was significantly higher than in positive control group. This means development in oocyte maturation after RA and FGF2 injection is considerable. Matos *et al*. (2007) demonstrated that FSH and FGF-2, alone or in combination, were the most effective treatment which increased the diameter of preantral follicles in caprine (16). This study strengthened Matos’s results. Different studies confirmed the concept of poor proliferation and low apoptosis in cystic follicles of rats and other animal models with PCOS phenotype. Therefore, low proliferation and apoptotic changes in the persistent follicle cysts are assumed as a part of PCO pathogenesis (17). Moreover, oxidative stress is a prevalent pathological event in PCOS and some studies suggested that RA as an anti-oxidant, has a protective effect on follicles. The oxidative stress in the follicle, which has been stimulated by apoptotic changes, can be stopped by RA (5). In this study, the synergistic effects of RA and FGF2 on increasing oocyte`s cytoplasmic diameter in PCOS-induced mice were observed. The oocytes contain RA receptors and they probably respond to exogenous or endogenous RA. Otherwise, in the presence of an oocyte cascade activator such as FGF2, they will respond to exogenous RA. 

This study revealed that a combination of FGF2 with RA has a synergistic effect on follicular development, as well as improvement of oocyte parameters such as cytoplasmic diameter and ZP thickness. This combination also increases the grade-A embryos. These results are consistent with Ghezelayagh *et al.* study. They described that FGF2 can control granulosa cells` development and theca cells` division. Thus, potentially can provoke angiogenesis and prevent steroidogenesis. Therefore, FGF2 can improve the permanence of primordial follicles (18). The results of the present study showed that the quality of mice embryos after combination treatment (with FGF2 and RA) increased, so the grade A embryos were higher in group 6 of the study groups. Another study described that FGF2 supplement *in vitro* resulted in the promotion of embryo cleavage and blastocyst development (19). Kumar *et al*. (2020) showed that expression of the FGF2 receptor in A-grade is higher than the C- and D-grade oocytes in buffalos (20).

It is supposed that dual treatment with RA and FGF2 has significant synergetic effects on oocyte development in PCOS-induced mice. Moreover, the results of this study showed that simultaneous injection of RA and FGF2 increased the number and maturity of ovarian follicles, increased the number of two-celled embryos, and increased the number of grade-A embryos in mice with PCOS. Jagtap *et al*. mentioned that previous studies on bovine trophoblast, have indicated the synergistic effect of FGF2 and RA during pre-implantation, on starting differentiation in the vertebrate axis. Also, based on their own study they concluded, that the presence of FGF2 will prevail over the RA effects on human stem cell differentiation (21). The results of this study are in the same direction as and support the concept of Jagtap’s study.

## Conclusion

FGF2, an endogenously produced oocyte competency factor by theca, granulosa, and cumulus cells throughout folliculogenesis, appears to have the potential to improve follicle growth, oocyte maturation parameters, and embryo development through supplementary administration. This study suggests that the combination of RA and fibroblast growth factor 2 can enhance the maturity of ovarian follicles, increase the number of two-celled embryos, and increase the number of grade-A embryos in mice with PCOS. Notably, the magnitude of this effect is greater when both substances are administered simultaneously. Consequently, further studies are needed to either validate or refute these findings, as this topic remains highly controversial. 

## Authors’ Contributions

A M, B N, RM M, and R MJ designed the study, collected data, performed experiments, and wrote and edited the manuscript. D SH, R A, and R SH conceived and designed the study, interpreted data, and revised the manuscript. D F analyzed data and revised the manuscript. Z S supervised the study and study design. All authors have read the final manuscript and approved the submission. I as the corresponding author confirm that the manuscript has been read and approved by all named authors, and there are no other people who meet the criteria for authorship that are no listed. I further confirm that the order of authors listed in the manuscript has been approved by all authors.

## Conflicts of Interest

The authors declare that they have no conflicts of interest.
